# Supplementary material for: Hierarchical Principal Components for Data-Driven Multiresolution fMRI Analyses
Source: Brain Sci. 2024 Mar 28;14(4):325. doi: 10.3390/brainsci14040325 (PMC11048444; doi:10.3390/brainsci14040325)
Supplement: Supplementary file 1 [file brainsci-14-00325-s001.zip › Supplementary Figure Legends.pdf]

Supplementary Figure Legends

Figure S1: Group average spatial maps for 5L2D levels 990-1023 from non-orthogonalized time series. For comparison with 5L2D spatial maps from orthogonalized time series, see main Figure 2. Levels 1021-1023 were flagged as merging unrelated variables by t-test (main Table 1, lower). As shown here, z-scores in spatial maps for these levels were very weak, suggesting statistical inference on these levels was correct.

Figure S2: Orthogonalized & non-orthogonalized spatial maps for experiment 5L2D. Top: true group-level spatial maps used in SimTB parameters. Bottom left: mean hierarchical principal component analysis (hPCA) back-reconstructed spatial maps from orthogonalized time series, following statistical filtering of levels. Bottom right: mean hPCA back-reconstructed spatial maps from non-orthogonalized time series.

Figure S3: Orthogonalized & non-orthogonalized spatial maps for experiment 3L3D. Top: true group-level spatial maps used in SimTB parameters. Bottom left: mean hierarchical principal component analysis (hPCA) back-reconstructed spatial maps from orthogonalized time series, following statistical filtering of levels. Bottom right: mean hPCA back-reconstructed spatial maps from non-orthogonalized time series.

Figure S4: Orthogonalized & non-orthogonalized spatial maps for experiment 3L3Ddirich. Top: true group-level spatial maps used in SimTB parameters. Bottom left: mean hierarchical principal component analysis (hPCA) back-reconstructed spatial

maps from orthogonalized time series, following statistical filtering of levels. Bottom right: mean hPCA back-reconstructed spatial maps from non-orthogonalized time series.

Figure S5: Accuracy of multi-model ICA applied to simulated fMRI datasets with hierarchical correlation matrices. Independent Component Analysis (ICA) was applied to all 3 experimental datasets with a varying model order (i.e., expected number of components), in order to test the dependence of ICA results on this parameter. Accuracy was measured as the correlation between ICA back-reconstructed spatial map and ground truth spatial map. In all three experiments and across all models tested ICA estimated one spatial scale with high accuracy, while poorly estimating spatial maps at different spatial scales. 3L3D: simulated hierarchy with 3 levels and 3 degree split at each level, 3L3Ddirich: hierarchy with 3 levels and 3 degree split and randomized block sizes, 5L2D: hierarchy with 5 levels and 2 degree split at each level.
