# Supplementary figures and images for: Hierarchical Principal Components for Data-Driven Multiresolution fMRI Analyses
Source: Brain Sci. 2024 Mar 28;14(4):325. doi: 10.3390/brainsci14040325 (PMC11048444; doi:10.3390/brainsci14040325)

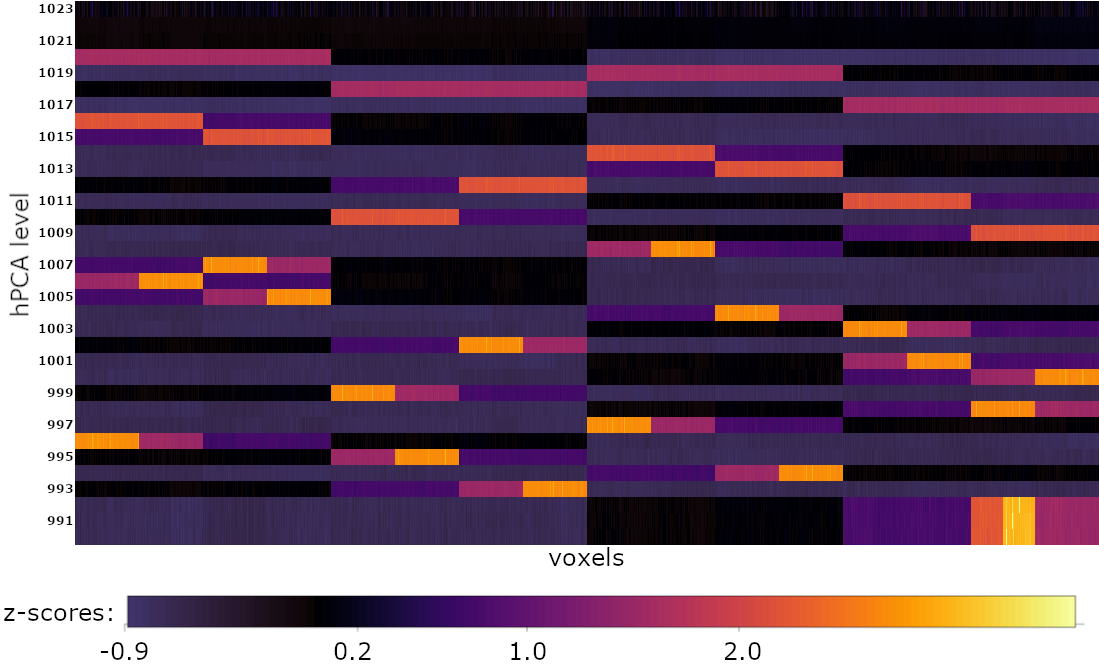

Supplement: Supplementary file 1 [file brainsci-14-00325-s001.zip › Figure S1.png]

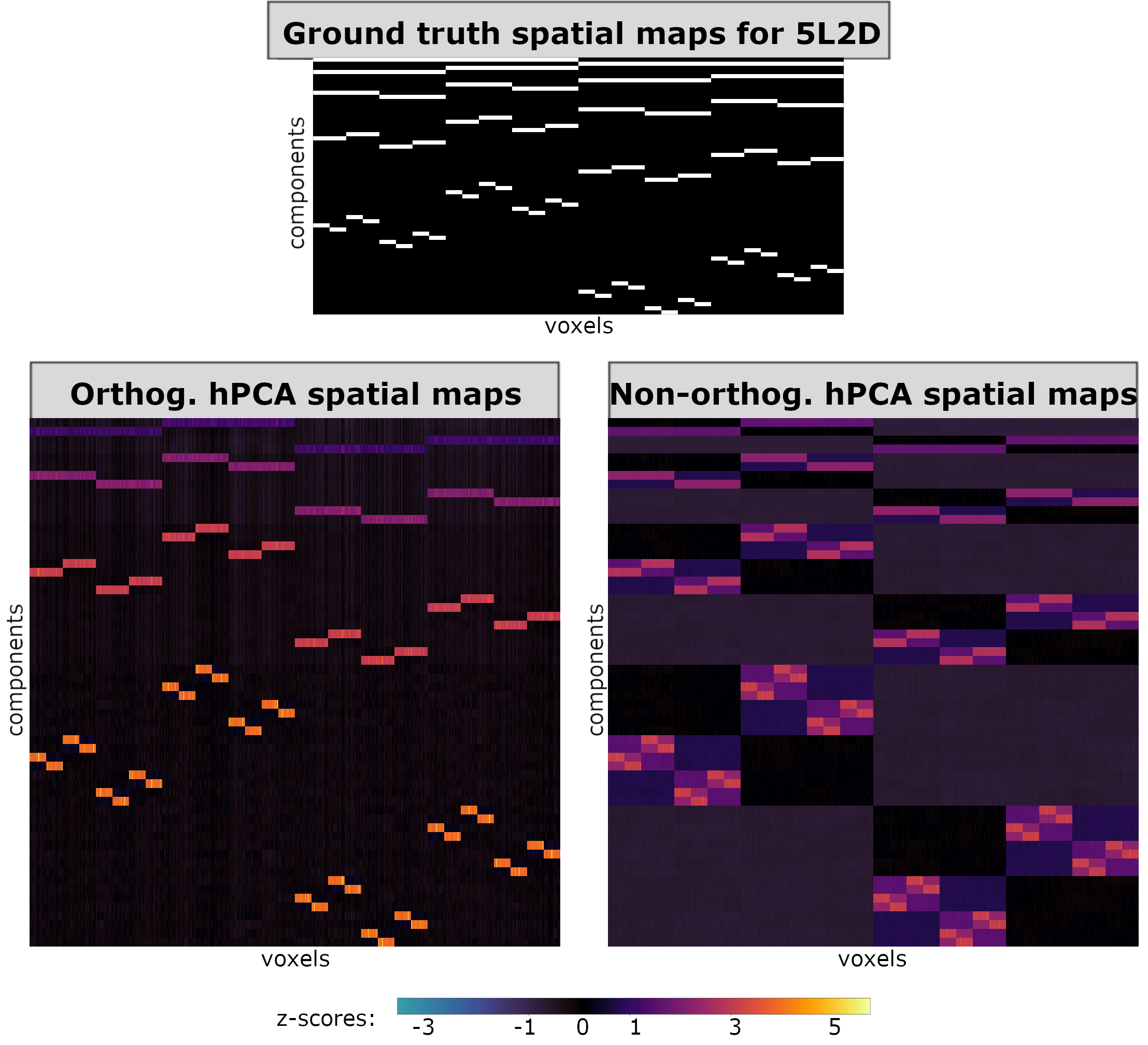

Supplement: Supplementary file 1 [file brainsci-14-00325-s001.zip › Figure S2.tiff]

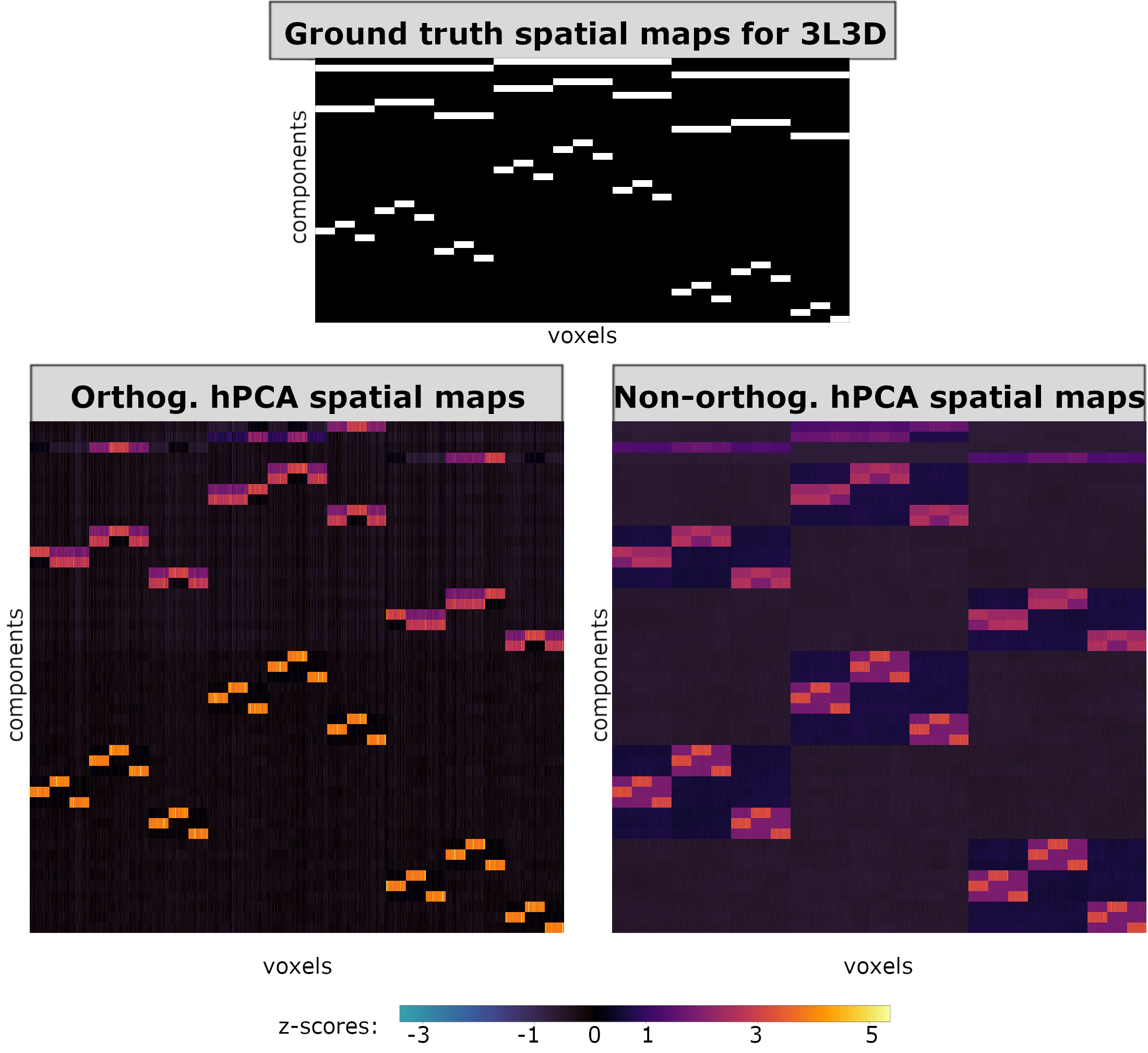

Supplement: Supplementary file 1 [file brainsci-14-00325-s001.zip › Figure S3.tiff]

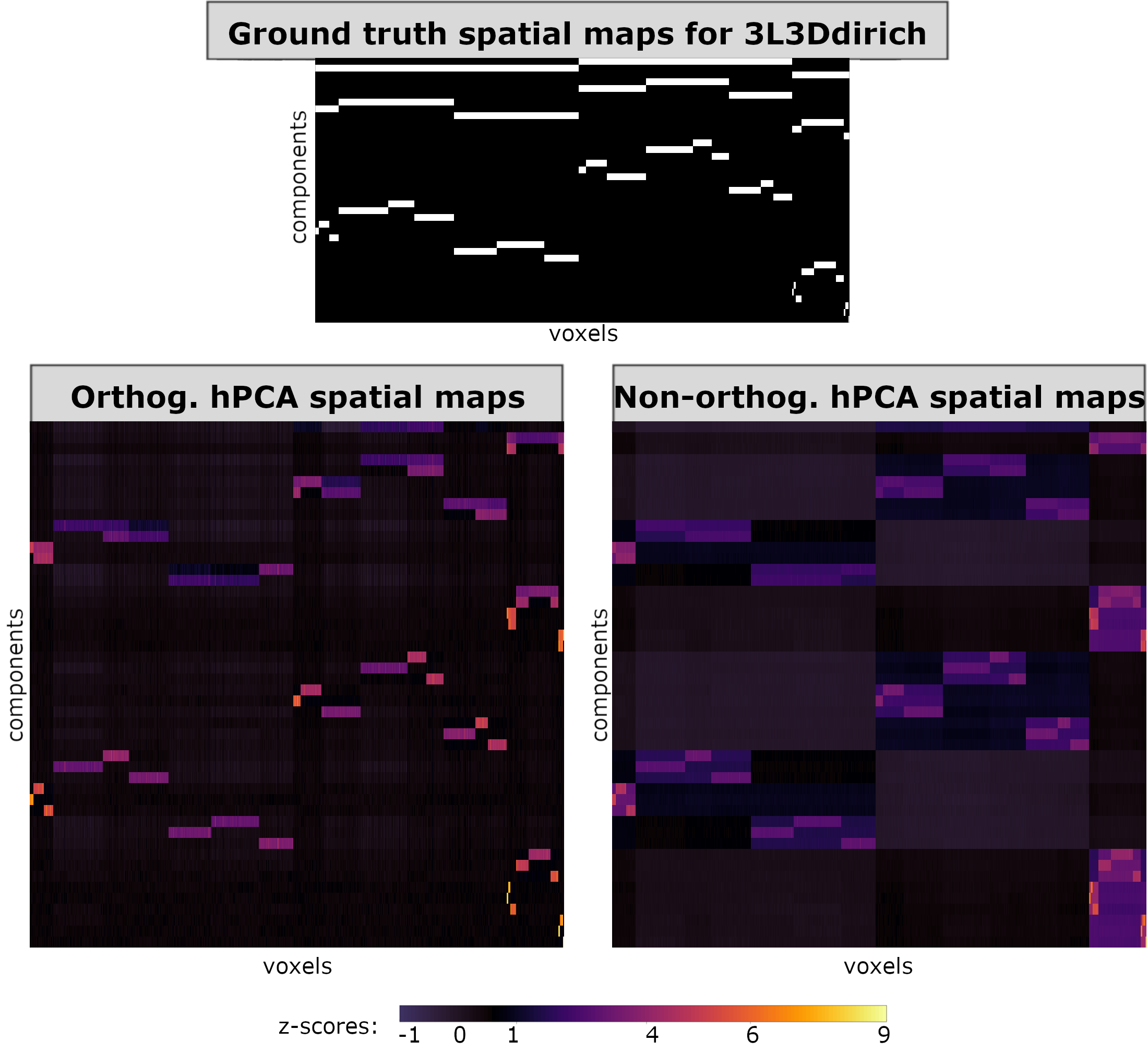

Supplement: Supplementary file 1 [file brainsci-14-00325-s001.zip › Figure S4.tiff]

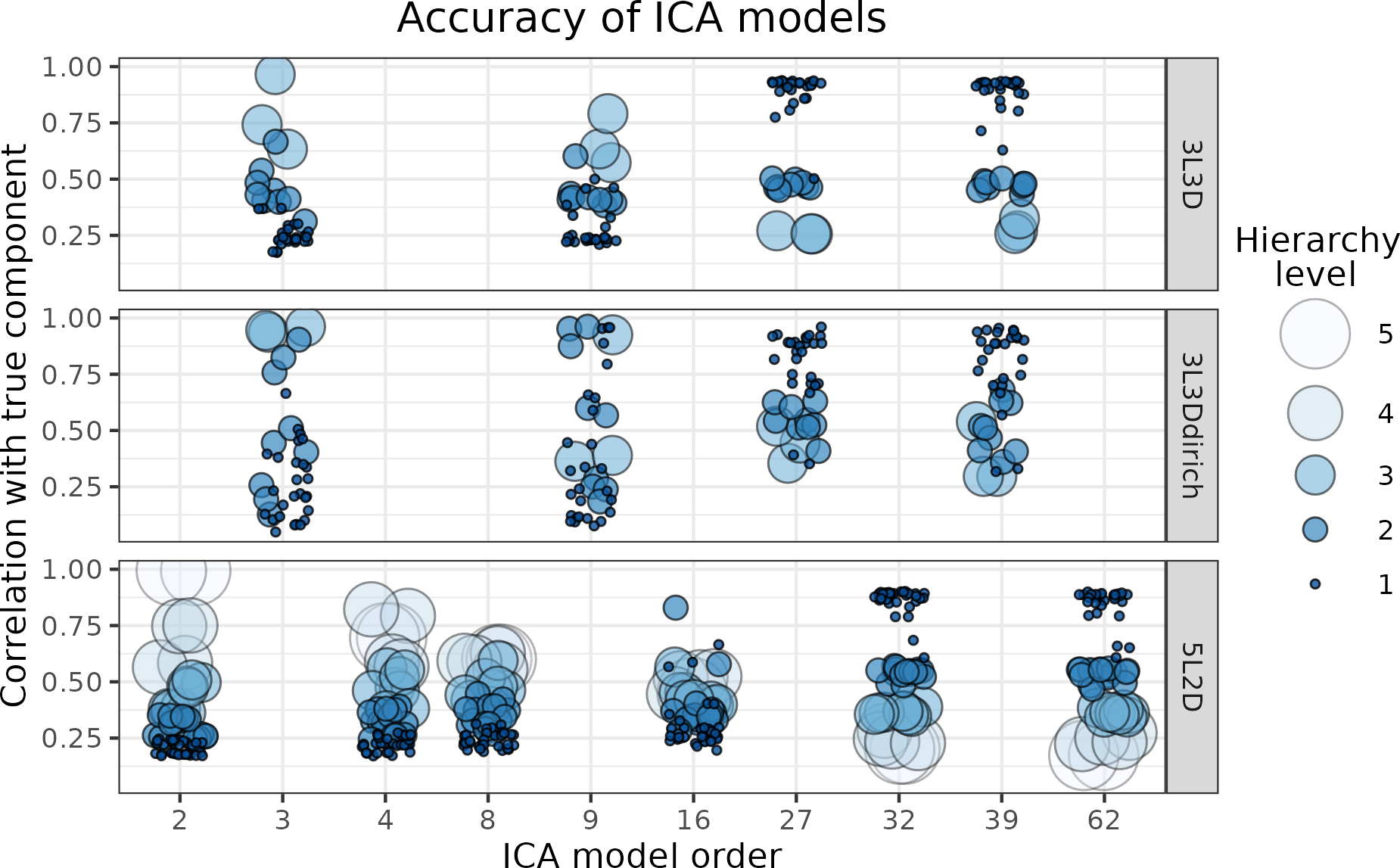

Supplement: Supplementary file 1 [file brainsci-14-00325-s001.zip › Figure S5.tiff]
